# Effect of age on the morphologies of the human Schlemm’s canal and trabecular meshwork measured with swept‑source optical coherence tomography

**DOI:** 10.1038/s41433-018-0148-6

**Published:** 2018-06-19

**Authors:** Zhiqi Chen, Jian Sun, Mu Li, Shiliang Liu, Liugui Chen, Sili Jing, Zhen Cai, Yan Xiang, Yinwei Song, Hong Zhang, Junming Wang

**Affiliations:** Department of Ophthalmology, Tongji Hospital, Tongji Medical College, Huazhong University of Science and Technology, Wuhan, 430030 China

## Abstract

**Purpose:**

We aimed to measure the sizes of Schlemm’s canal (SC) and the trabecular meshwork (TM) in healthy individuals and to evaluate variations with age from childhood to old age by using swept-source optical coherence tomography (OCT).

**Methods:**

Anterior chamber angle imaging of the superior, inferior, nasal, and temporal regions of the right and left eyes was performed with swept-source OCT. The diameter and area of SC and TM width and thickness were measured manually from OCT images.

**Results:**

A total of 114 healthy individuals were enrolled and included 48 male subjects and 66 female subjects; their ages ranged from 7 to 83 years. Both the SC diameter and area in the four quadrants decreased significantly with aging (*P* < 0.001) and were wider in the nasal and temporal quadrants compared with the superior and inferior quadrants. Changes in SC size showed significant positive correlations with axial length (AL) and anterior chamber depth (*P* < 0.001). There was a significant positive association between age and TM thickness in the nasal and temporal quadrants (*P* < 0.05). The inferior quadrant TM width was the widest among the quadrants. The superior quadrant TM thickness was the thinnest among the quadrants. Changes in TM thickness in the nasal and temporal quadrants showed a significant negative correlation with AL (*P* < 0.05). There was no statistically significant correlation in SC and TM parameters with central corneal thickness, intraocular pressure, sex, or right or left eye (*P* > 0.05).

**Conclusions:**

With aging, the SC diameter and area became smaller, TM thickness increased, and TM width seemed to remain constant. Measurements of the sizes of SC and the TM with swept-source OCT could assist in clinical assessments and treatment planning for glaucoma.

Elevated intraocular pressure (IOP) is the most important risk factor for glaucoma [1], with aqueous outflow obstruction playing the dominant role in IOP regulation [2]. Aqueous humor is secreted by the ciliary body; 75% to 80% flows out through the conventional trabecular meshwork (TM)–Schlemm’s canal (SC) pathway [3]. As the main resistance points in the eye’s aqueous pathway, changes in the TM and SC are related to outflow facility and can cause IOP changes [4–6]. In our previous study by ultrasound biomicroscopy examinations [7], we reported that patients with primary open-angle glaucoma had a less observable SC, smaller SC diameter, and decreased TM thickness than normal individuals; the underlying reasons require further study. While age is an important risk factor in glaucoma development in vivo [8], the detailed effects of age on SC and the TM from childhood to old age remain unknown.

Optical coherence tomography (OCT) is a non-invasive technique that can detect microstructural changes in the anterior chamber angle in vivo. With advances in the acquisition speed and imaging resolution, both SC and the TM can be identified continuously and dynamically [9, 10]. Therefore, in this study, we attempted to observe age-related changes in human SC, TM, and anterior chamber parameters in normal eyes from childhood to old age using swept-source OCT.

## Materials and methods

Participants were enrolled from the Department of Ophthalmology, Tongji Hospital, Tongji Medical College, Huazhong University of Science and Technology, Wuhan, China from November 2016 to February 2017. The research was approved by the Institutional Review Board and followed the tenets of the Declaration of Helsinki. All subjects signed informed consent forms. For subjects under the age of 18 years, informed consent was also required from the parents in these cases.

Healthy volunteers were recruited from the outpatient department of Ophthalmology, Tongji Hospital, Tongji Medical College, Huazhong University of Science and Technology. Subjects under the age of 18 years came to hospital because of low myopia. All subjects underwent complete ophthalmologic examinations including best-corrected visual acuity (BCVA), refractive error, IOP (non-contact tonometer; NIDEK RT-2100; NIDEK, CO., LTD, Gamagori, Japan), central corneal thickness (CCT), and axial length (AL) as well as a slit-lamp examination and fundus examination. AL measurement was performed using an IOLMaster (Carl Zeiss, Inc., Jena, Germany). Healthy volunteers without ocular diseases with a BCVA of 20/25 or better, a refractive error between +3.0 and −6.0 D, IOP between 10 and 21 mm Hg, and normal optic disc and retina were included. The clinical trial registry number was ChiCTR-ROC-16008832.

### OCT data acquisition and processing

In addition to an ocular examination, all participants underwent examinations with swept-source OCT (CASIA SS-1000; Tomey Corporation, Nagoya, Japan), which is specifically designed for anterior segment imaging using a 1310 nm wavelength, scan speed of 30,000 A-scans per second, and axial resolution of less than 10 µm. The eyes were imaged in a dark room by the same examiner (JS). For the children, because it is difficult to stare up or down steadily, the scans were performed independently for the nasal and temporal quadrants (3 o’clock and 9 o’clock positions). For the adults, the superior and inferior quadrants (6 o’clock and 12 o’clock positions) were also observed. The three-dimensional-angle high-definition protocol was a volumetric scan (dimensions, a raster of 64 B-scans each with 512 A-scans over 8 mm). Seated subjects were instructed to stare at one of four peripheral fixation lights to ensure that the iridocorneal angle was centered in the instrument’s field of view. If necessary, the operator assisted in opening the eyelid when examining the superior and inferior quadrants while taking care to avoid pressing on the eye. Scans of each site were repeated three times, and three images of each site centered at the 3, 6, 9, and 12 o’clock positions were chosen for the final analysis.

**Fig. 1:**
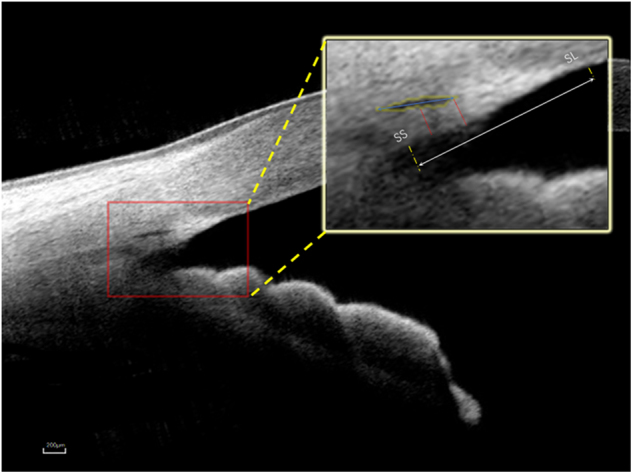
Example of Schlemm’ s canal (SC) and trabecular meshwork (TM) measurements made using the swept‑source optical coherence tomography. The black oval space shows SC. The SC diameter: from the anterior to the posterior endpoint of SC (blue arrow). The SC area: drawn freehand and depicted as the area surrounded by the outline of the SC (yellow outline). The TM width: the distance between the scleral spur and Schwalbe’s line (white arrow). The TM thickness: two measurements made at the anterior endpoint of SC and halfway down SC (red lines), the average of them is calculated as TM thickness

The SC was defined as observable when a thin, black, lucent space on the images. The percentage of sections with an observable SC was defined as follows: eyes with a completely observable SC/the total number of eyes × 100. For each image, in individuals with SC visibility, the SC diameter and area and TM width and thickness were assessed and then quantified manually using ImageJ software (http://imagej.nih.gov/ij/; provided in the public domain by the National Institutes of Health, Bethesda, MD, USA) by an independent masked observer (ZC). The scleral spurwas defined as the point between the TM and the ciliary body. The SC diameter was measured from the posterior to anterior SC endpoints (Fig. 1). The SC area was drawn freehand and depicted as the area surrounded by the outline of the SC (Fig. 1). The TM width was defined as the distance between the scleral spur and Schwalbe’s line (Fig. 1). Each TM thickness measurement was made perpendicular to the inner layer of the meshwork. The TM thickness was calculated as the average of two measurements made at the anterior endpoint of SC and halfway down SC (Fig. 1). The anterior chamber depth (ACD) was defined as the perpendicular distance between the corneal endothelium at the corneal apex and the anterior lens surface. The central corneal thickness (CCT) was also measured using a CASIA SS-1000 OCT system. To measure intraobserver repeatability, 30 eyes were randomly chosen. The images of these eyes were evaluated by a single masked observer (ZC) on two separate times at an interval of 3 days, and agreement between the two observations was analyzed. To measure interobserver reproducibility, the same images were evaluated by two independent and well-trained observers (ZC and ML), and the agreement between them was determined. The intraclass correlation coefficients were calculated using a two-way mixed effect model.

### Statistical analyses

All analyses were performed using the SPSS software package version 19.0(SPSS, Inc., Chicago, IL, USA). Data are presented as the mean ± standard deviation where applicable. The Kruskal–Wallis *H*-test was used for comparisons of different parameters among the four quadrants in the same groups. Student’s *t*-test was used to analyze differences between the right and left eyes and between male and female subjects. Nonparametric Spearman's correlation analyses were performed to examine relationships between age and SC and TM parameters statistically. All tests were two-tailed and statistical significance was defined as *P* < 0.05.

## Results

In total, the study included 114 normal subjects (48 male subjects and 66 female subjects; age range, 7–83 years). The subjects were divided into four groups according to age: A, 20 subjects (7–14 years); B, 36 subjects (21–39 years); C, 37 subjects (40–59 years); and D, 21 subjects (60–83 years). The characteristics of the enrolled subjects are summarized in Table 1. The measurements of SC and TM parameters showed excellent repeatability and reproducibility with the intraclass correlation coefficients of >0.9 (all *P* < 0.01).

**Table 1:** Subject and ocular characteristics

	Age group				Total subjects
	7–14 y	20–39 y	40–59 y	60–83 y	
*N*	20	36	37	21	114
Male/Female	11/9	19/17	11/26	7/14	48/66
Mean age (y)	10 ± 2.3	27.1 ± 3.8	48.5 ± 4.4	66.6 ± 7.1	38.3 ± 19.6

	IOP (mm Hg)	CCT (μm)	AL (mm)	ACD (mm)	Refraction (D)
Right eyes	15.6 ± 2.5	537.1 ± 28.9	23.8 ± 1.1	2.89 ± 0.4	-1.27 ± 1.78
Left eyes	15.7 ± 2.5	537.7 ± 28.7	23.7 ± 1.1	2.86 ± 0.5	-1.06 ± 1.64

*CCT* central corneal thickness, *IOP* intraocular pressure, *AL* axial length, *ACD* anterior chamber depth

Data are presented as mean  ±  SD

**Fig. 2:**
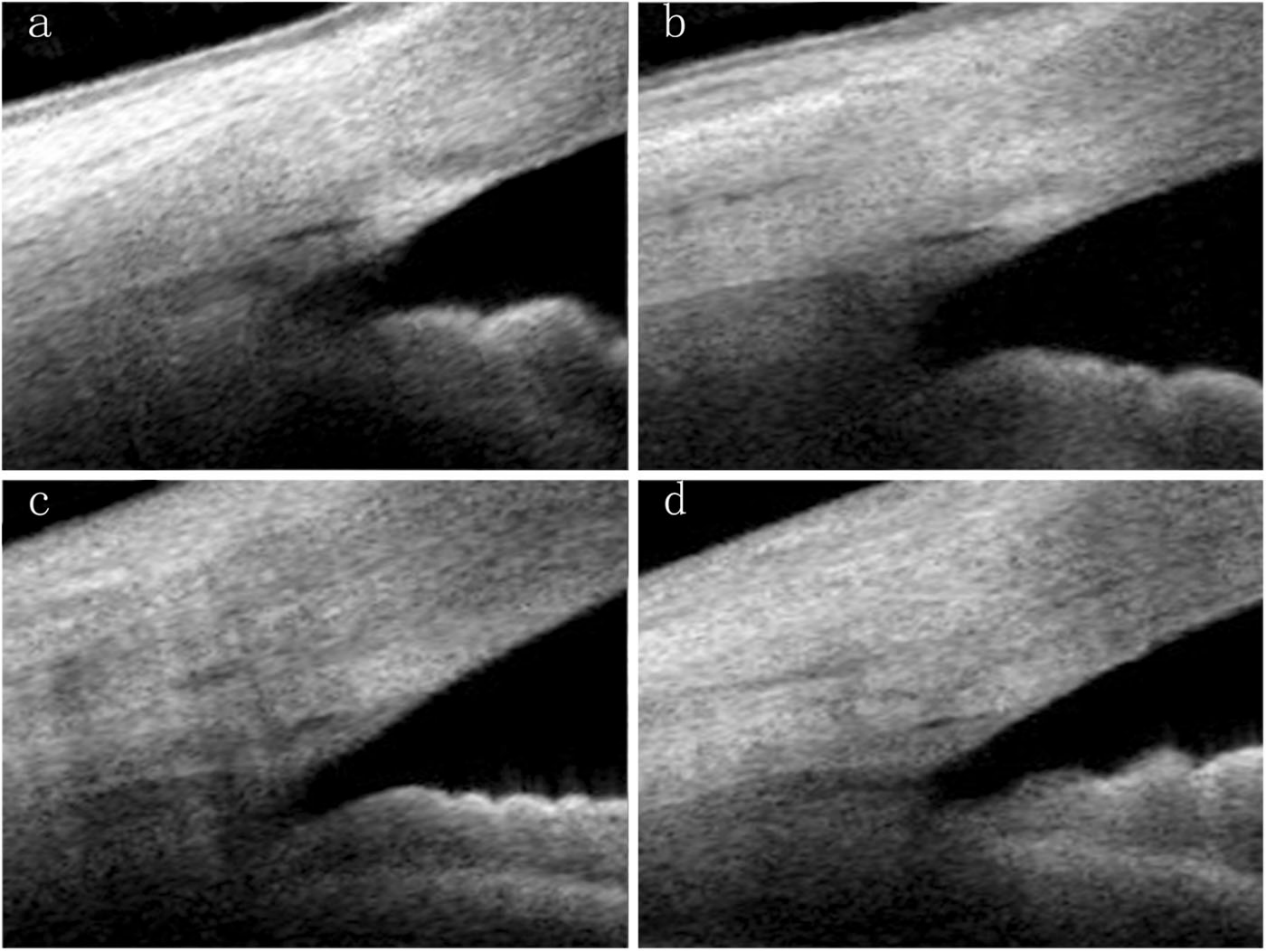
The Schlemm’s canal (SC) and the trabecular meshwork (TM) morphology manifestation in each age group. **a** A 8-year-old female subject. **b** A 34-year-old female subject. **c** A 48-year-old female subject. **d** A 75-year-old female subject. From (**a** to **d**) with aging, the SC diameter and area became smaller, and TM thickness increased

**Fig. 3:**
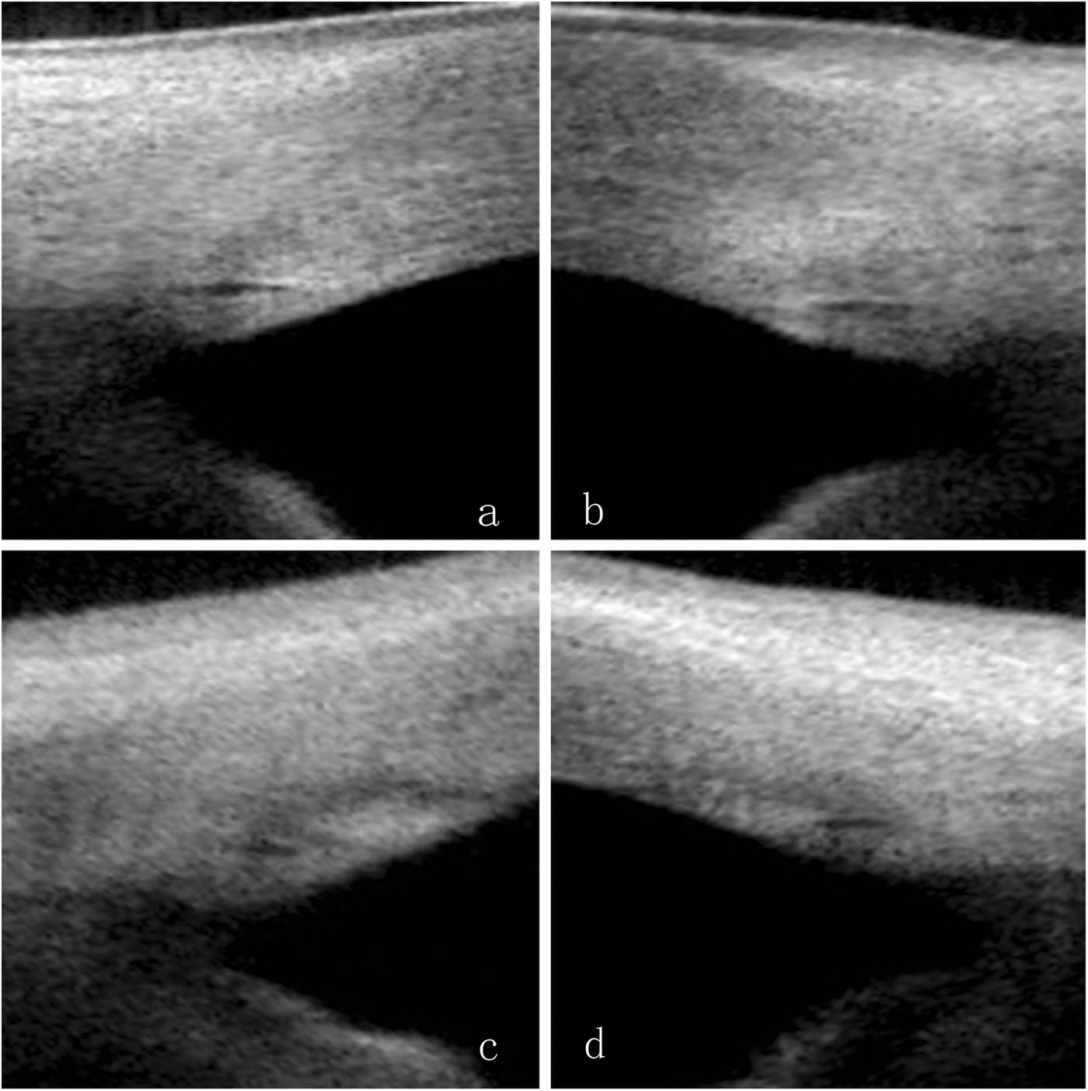
Quadrant comparisons of a 26-year-old male subject indicated that both the Schlemm’s canal (SC) diameter and area were wider in the nasal (**a**) and temporal (**b**) quadrants compared with the inferior (**c**) and superior (**d**) quadrants

In the children’s group (7–14 years), SC was observable in 100% of the sections, which was significantly higher than in the other groups: 95.1% in group B (21–39 years), 88.3% in group C (40–59 years), and 80.6% in group D (60–83 years). In all individuals with SC visibility, the SC diameter varied from 50.8 μm to 393.5 μm in the different quadrants of the eye and the SC area ranged from 835.3 μm ^2^ to 17,727.1 μm^2^. When compiled according to the four age categories, the greatest diameter was measured in the children’s group (Fig. 2a); a clear decline in the mean SC diameter in the four quadrants was evident with age, and the linear correlation analysis indicated a significant age-dependent tendency (*P* < 0.001) (Table 2a, Fig. 2). We also demonstrated that age had the same effects on the SC area (Table 2b, Fig. 2). The changes in SC showed significant correlations with AL and ACD (*P* < 0.001), while other factors such as CCT and IOP did not vary with any SC parameter (*P* > 0.05). Quadrant comparisons indicated that both the SC diameter and area were wider in the nasal and temporal quadrants compared with the inferior and superior quadrants (Fig. 3) (*P* < 0.001).

**Table 2a:** Mean SC diameter (μm) in four quadrants in each age group and correlation with age

Quadrant	Total subjects	Age group				Correlation with age	
		7–14 y	20–39 y	40–59 y	60–83 y	*r*	*P*
Right eyes							
Nasal	152.69 ± 68.7	214.6 ± 70.99	158.02 ± 69.99	127.82 ± 50.16	119.13 ± 45.03	−0.511	0.000^a^
Temporal	159.69 ± 61.26	174.07 ± 73.46	187.44 ± 58.48	143.79 ± 54.6	122.42 ± 32.6	−0.31	0.001^a^
Superior	137.52 ± 69.3		167.19 ± 86.04	121.54 ± 47.96	104.08 ± 32.23	−0.344	0.002^a^
Inferior	133.43 ± 67.85		152.45 ± 75.3	126.44 ± 67.52	102.59 ± 27.07	−0.515	0.000^a^
Left eyes							
Nasal	159.88 ± 69.43	225.38 ± 68.16	173.35 ± 64.68	120.21 ± 39.52	117.95 ± 48.55	−0.537	0.000^a^
Temporal	161.11 ± 74.95	221.18 ± 94.49	181.24 ± 61.3	125.57 ± 43.76	102.79 ± 42.64	−0.583	0.000^a^
Superior	137.68 ± 53.11		156.48 ± 57.74	119.63 ± 36.77	124.34 ± 58.27	−0.29	0.015^a^
Inferior	130.74 ± 52.54		149.58 ± 59.18	105.21 ± 37.47	127.3 ± 32.78	−0.205	0.098

Data are presented as mean  ±  SD

^a^Correlation is significant at *P* < 0.05 (Spearman’s test, *r*: correlation coefficient)

**Table 2b:** Mean SC area (μm^2^) in four quadrants in each age group and correlation with age

Quadrant	Total subjects	Age group				Correlation with age	
		7–14 y	20–39 y	40–59 y	60–83 y	*r*	*P*
Nasal	5176.84 ± 3273.69	9021.98 ± 2708.52	5656.19 ± 3237.39	3571.23 ± 1908.05	2916.01 ± 1377.04	−0.664	0.000^a^
Temporal	4802.13 ± 2420.95	6141.01 ± 2601.81	5779.04 ± 2178.25	4046.69 ± 2039	2989.04 ± 1648.06	−0.492	0.000^a^
Superior	3474.6 ± 1957.14		4555.97 ± 2420.03	2769.13 ± 1140.86	2538.18 ± 569.84	−0.433	0.000^a^
Inferior	3568.65 ± 1935.29		4621.72 ± 2072.96	2986.04 ± 1488.16	2251.66 ± 853.58	−0.515	0.000^a^
Left eyes							
Nasal	5465.58 ± 4484.34	11060.82 ± 5486.16	6078.31 ± 3265.48	3515.15 ± 1684.05	2935.6 ± 1696.55	−0.629	0.000^a^
Temporal	5558.94 ± 4255.11	9620.28 ± 6093.91	6257.83 ± 3237.93	3500.33 ± 1629.2	2493.06 ± 1392.47	−0.669	0.000^a^
Superior	3777.87 ± 1779.02		4441.98 ± 1748.95	3331.99 ± 1620.99	2790.19 ± 1581.23	−0.409	0.000^a^
Inferior	3981.84 ± 2147.53		5019.78 ± 2265.69	2917.1 ± 1413.82	3005.51 ± 1504.52	−0.537	0.000^a^

Data are presented as mean  ±  SD

^a^Correlation is significant at *P* < 0.05 (Spearman’s test, *r*: correlation coefficient)

The TM width ranged from 476.9 μm to 1218.8 μm. The inferior quadrant TM width was the widest among the quadrants (Table 2c). The TM thickness ranged from 43.4 μm to 284.4 μm. In Table 2d, the superior quadrant TM thickness was the thinnest among the quadrants. There was a significant positive association between TM thicknesses in the nasal and temporal quadrants and age (*P* < 0.05). Changes in TM thickness in the nasal and temporal quadrants showed significant negative correlations with AL (*P* < 0.05), while other factors such as CCT, ACD, and IOP did not vary with any TM parameter (*P* > 0.05).

**Table 2c:** Mean TM width (μm) in four quadrants in each age group and correlation with age

Quadrant	Total subjects	Age group				Correlation with age	
		7–14 y	20–39 y	40–59 y	60–83 y	*r*	*P*
Right eyes							
Nasal	778.39 ± 107.61	705.28 ± 97.93	811.07 ± 108.14	794.81 ± 89.2	763.86 ± 115.65	0.138	0.144
Temporal	769.91 ± 95.82	719.26 ± 74.62	786.24 ± 76.81	795.13 ± 110.84	746.92 ± 97.78	0.065	0.494
Superior	763.14 ± 118.92		799.67 ± 107.12	764.88 ± 121.99	686.67 ± 103.91	−0.323	0.002^a^
Inferior	838.54 ± 126.35		845.76 ± 113.04	853.23 ± 135.4	794.72 ± 130.09	−0.126	0.236
Left eyes							
Nasal	782.79 ± 106.62	714.87 ± 101.37	798.73 ± 102.32	810.93 ± 94.61	776.34 ± 115.51	0.217	0.025^a^
Temporal	784.93 ± 104.52	723.49 ± 90.34	825.63 ± 95.75	799.44 ± 87.36	746.82 ± 124.45	0.037	0.707
Superior	801.61 ± 127.75		830.23 ± 102.98	796.42 ± 133.08	745.57 ± 155.18	−0.213	0.057
Inferior	870.1 ± 109.05		978.37 ± 95.19	869.02 ± 112.46	852.31 ± 136.51	−0.075	0.509

Data are presented as mean  ±  SD

^a^Correlation is significant at *P* < 0.05 (Spearman’s test, *r*: correlation coefficient)

**Table 2d:** Mean TM thickness (μm) in four quadrants in each age group and correlation with age

Quadrant	Total subjects	Age group				Correlation with age	
		7–14 y	20–39 y	40–59 y	60–83 y	*r*	*P*
Right eyes							
Nasal	125.23 ± 44.95	103.03 ± 31.95	119.52 ± 30.64	130.71 ± 40.81	150.94 ± 70.13	0.256	0.008^a^
Temporal	116.79 ± 28.98	114.63 ± 40.32	114.94 ± 30.7	114.79 ± 19.6	125.67 ± 26.19	0.191	0.046^a^
Superior	109.25 ± 36.01		105.78 ± 34.49	111.38 ± 36.93	112.56 ± 39.29	0.039	0.735
Inferior	121.95 ± 38.21		125.98 ± 39.57	113.76 ± 35.71	128.8 ± 39.64	−0.106	0.364
Left eyes							
Nasal	112.33 ± 34.75	93.71 ± 24.3	110.73 ± 32.45	122.51 ± 40.41	121.28 ± 32.71	0.229	0.023^a^
Temporal	114.32 ± 34.46	106.32 ± 38.75	110.27 ± 32.9	122.09 ± 35.72	119.45 ± 28.38	0.194	0.055
Superior	99.73 ± 23.02		98 ± 24.42	100.58 ± 21.24	103.14 ± 24.78	0.036	0.77
Inferior	127.61 ± 44.56		125.81 ± 41.97	125.9 ± 48.08	124.99 ± 61.54	−0.006	0.959

Data are presented as mean  ±  SD

^a^Correlation is significant at *P* < 0.05 (Spearman’s test, *r*: correlation coefficient)

Additionally, a negative correlation was found between TM thickness and SC size (SC diameter: *r* = −0.083, *P* = 0.028; SC area: *r* = −0.13, *P* = 0.001), but the *R* coefficients were very weak. Otherwise, differences in measurements of SC and TM parameters were not statistically significant between the right and left eyes or between the sexes.

## Discussion

Our study demonstrated that both the SC diameter and area decreased significantly with age and TM thickness increased with age. To our knowledge, this is the first in vivo study to report significant correlations between SC and TM biometric parameters and age in normal individuals (aged 7–83 years) with swept-source OCT and provides detailed physiological data for the four quadrants.

A previous study [11] on SC development in human fetal eyes reported that an incomplete SC could be observed in some sections of the limbal region at 27 weeks of gestation and that the canal was complete in most sections at about 40 weeks of gestation. Until now, no data have been reported on children. Our study filled in this gap and our results show that SC could be observed easily in 100% of sections from children aged 7 to 14 years, with a especially distinctive full, black, and lucent space on the images, and the SC size in the children’s group was the largest among the groups and revealed a well-filled and healthy outflow status in children’s SCs.

In our current study, the SC diameter and area were decreased significantly with aging, which was in partial agreement with previous histopathologic SC studies performed on donor eyes [12, 13], and SC was observable in only 80.6% of eyes from the older group (60–83 years). We speculate that the decreased SC size and lower SC detection rates in older subjects might be caused by several reasons. First, the main SC structures affected by aging included a reduced density of giant vacuoles and diminished intracellular and intercellular pore populations [13, 14]; SC degeneration itself may lead to SC shrinkage. Second, age-related loss in ciliary muscle movement and an altered limbal corneoscleral contour [15, 16] may lead to diminished traction on SC. Third, as aqueous production decreases with age [17], a presumably lower flow into SC will result in a reduced SC size.

We found that TM thickness in the nasal and temporal quadrants was positively correlated with age; previous studies have shown that the elasticity of the TM is decreased with age [18]. Thickening of the TM elastic fibers and increased extracellular matrix would lead to an increased TM thickness in older individuals, which may decrease drainage of the aqueous through pores of the cribriform net and lead to increased resistance to aqueous outflow. We speculate that this might be one reason that older individuals are more likely to develop glaucoma. Otherwise, no significant correlation between TM width and age was observed in our study, which is consistent with previous reports [19, 20], and because we defined the TM width as the distance between the scleral spur and Schwalbe’s line, these anatomical landmarks as TM boundaries might not vary with increasing age.

Our results suggest that SC and TM parameters varied in the different quadrants of the eye. Both SC diameter and area were greater in the nasal and temporal quadrants than in the inferior and superior quadrants, which may suggest preferential nasal and temporal drainage in the normal healthy eye, and may suggest that interventions designed to enhance outflow on the nasal and temporal sides are preferable. Researchers reported that nasal SC areas were significantly larger than temporal ones [21], while in our study, there was no significant difference between the nasal and temporal quadrants with respect to SC diameter or area, which was compatible with previous description [22, 23]. Referring to the TM parameters in the four quadrants, the inferior quadrant TM width was the widest among the quadrants; this result was exactly the same as that in previous study [24], which proved the reliability of our results. Otherwise, the superior quadrant TM thickness was the thinnest among the quadrants, which may be the result of the effects of gravity on aqueous humor metabolites.

AL exhibited a statistically significant association with SC size and TM thickness; a shorter AL was associated with a smaller SC size and thicker TM, which provides evidence for observations that individuals with a short AL may be more likely to suffer from angle-closure glaucoma [25].

The present study has certain limitations. First, in order to expose a quadrant, the subject should stare in the opposite direction, which is the same as the position used during a dynamic gonioscopic examination, not the primary ocular position. The impact of these ocular positions on the SC and TM sizes require further study. Second, in our study, all measurements were obtained from 8:00 AM to 12:00 AM; 24 h variations in the SC and TM were not observed. Third, because it is difficult for the children to stare up or down steadily, OCT data from the superior and inferior quadrants were absent in the children’s group.

With aging, the SC size decreased and TM became thicker, and whether such aqueous outflow changes might affect the IOP and increase the risk of glaucoma in the long term still require verification.

### Summary

#### What was known before

- Previous histopathologic Schlemm’s canal (SC) studies performed on donor eyes showed that the main schlemm’s canal structures affected by aging included a reduced density of giant vacuoles and diminished intracellular and intercellular pore populations.
- Previous studies have shown that the elasticity of the trabecular meshwork (TM) is decreased with age.

#### What this study adds

- With aging in vivo, the SC diameter and area became smaller, TM thickness increased, and TM width seemed to remain constant.
- Both the SC diameter and area were wider in the nasal and temporal quadrants compared with the inferior and superior quadrants.
- The inferior quadrant TM width was the widest among the quadrants. The superior quadrant TM thickness was the thinnest among the quadrants.
